# A catalog of the genetic causes of hereditary angioedema in the Canary Islands (Spain)

**DOI:** 10.3389/fimmu.2022.997148

**Published:** 2022-09-20

**Authors:** Alejandro Mendoza-Alvarez, Eva Tosco-Herrera, Adrian Muñoz-Barrera, Luis A. Rubio-Rodríguez, Aitana Alonso-Gonzalez, Almudena Corrales, Antonio Iñigo-Campos, Lourdes Almeida-Quintana, Elena Martin-Fernandez, Dara Martinez-Beltran, Eva Perez-Rodriguez, Ariel Callero, Jose C. Garcia-Robaina, Rafaela González-Montelongo, Itahisa Marcelino-Rodriguez, Jose M. Lorenzo-Salazar, Carlos Flores

**Affiliations:** ^1^ Research Unit, Hospital Universitario Nuestra Señora de Candelaria, Santa Cruz de Tenerife, Spain; ^2^ Genomics Division, Instituto Tecnológico y de Energías Renovables, Santa Cruz de Tenerife, Spain; ^3^ Universidad de Santiago de Compostela, Santiago de Compostela, Spain; ^4^ CIBER de Enfermedades Respiratorias, Instituto de Salud Carlos III, Madrid, Spain; ^5^ Allergy Service, Hospital Universitario de Gran Canaria Dr. Negrín, Las Palmas de Gran Canaria, Spain; ^6^ Allergy Service, Hospital Universitario Dr. Molina Orosa, Las Palmas de Gran Canaria, Spain; ^7^ Allergy Service, Hospital Universitario Insular-Materno Infantil, Las Palmas de Gran Canaria, Spain; ^8^ Allergy Service, Hospital Universitario Nuestra Señora de Candelaria, Santa Cruz de Tenerife, Spain; ^9^ Public Health and Preventive Medicine Area, Universidad de La Laguna, Santa Cruz de Tenerife, Spain; ^10^ Facultad de Ciencias de la Salud, Universidad Fernando Pessoa Canarias, Las Palmas de Gran Canaria, Spain

**Keywords:** hereditary angioedema, genetic cause, rare disease, variant interpretation, precision medicine

## Abstract

Hereditary angioedema (HAE) is a rare disease where known causes involve C1 inhibitor dysfunction or dysregulation of the kinin cascade. The updated HAE management guidelines recommend performing genetic tests to reach a precise diagnosis. Unfortunately, genetic tests are still uncommon in the diagnosis routine. Here, we characterized for the first time the genetic causes of HAE in affected families from the Canary Islands (Spain). Whole-exome sequencing data was obtained from 41 affected patients and unaffected relatives from 29 unrelated families identified in the archipelago. The Hereditary Angioedema Database Annotation (HADA) tool was used for pathogenicity classification and causal variant prioritization among the genes known to cause HAE. Manual reclassification of prioritized variants was used in those families lacking known causal variants. We detected a total of eight different variants causing HAE in this patient series, affecting essentially *SERPING1* and *F12* genes, one of them being a novel *SERPING1* variant (c.686-12A>G) with a predicted splicing effect which was reclassified as likely pathogenic in one family. Altogether, the diagnostic yield by assessing previously reported causal genes and considering variant reclassifications according to the American College of Medical Genetics guidelines reached 66.7% (95% Confidence Interval [CI]: 30.1-91.0) in families with more than one affected member and 10.0% (95% CI: 1.8-33.1) among cases without family information for the disease. Despite the genetic causes of many patients remain to be identified, our results reinforce the need of genetic tests as first-tier diagnostic tool in this disease, as recommended by the international WAO/EAACI guidelines for the management of HAE.

## Introduction

Hereditary angioedema (HAE) is a rare genetic dominant condition with incomplete penetrance characterized by recurrent swellings (edema) affecting the skin, internal organs, mucosa or the upper airways (1, 2). Where genetic pathogenesis is known, symptoms are caused by dysfunction of the C1 esterase inhibitor (C1-INH) or dysregulation of the kinin cascade, leading to bradykinin release and resulting in inflammation episodes. Bradykinin is a vasoactive peptide and the main activator of the bradikinin receptor B2 expressed in endothelial cells (3) whose activation leads to increased vascular permeability and edema, causing the HAE symptoms (4). HAE attacks can turn into a life-threatening episode if the edema develops in the laryngeal track, which leads to the obstruction of the upper respiratory airways (5). HAE attacks are unpredictable and often develop in response to triggering factors such as mental stress (6), use of contraception hormones (7), infections (8), injuries or surgery interventions (9), and weather changes (6), among others. HAE prevalence has been estimated in over 1:50,000 worldwide (10) and has been reported in all ethnic groups (11).

This genetic condition is typically divided in two main groups attending to the C1-INH plasmatic levels. The most frequent form (85% of the cases described) is caused by decreased levels of C1-INH (11, 12), also known as HAE-C1-INH of type I. The rest of cases are found either with normal levels of non-functional C1-INH protein (also known as HAE-C1-INH of type II) or with normal levels of a functional C1-INH protein (HAE-nC1-INH). At the moment, more than 450 disease causing variants have been reported in *SERPING1* gene leading to HAE-C1-INH forms (11, 13–15). However, causal variants affecting other genes of the bradykinin pathway underlie HAE-nC1-INH cases (11).

The first genetic study in HAE families with normal levels of C1-INH detected a mutation in the exon nine of *F12* (16). FXII activation increases the bradykinin accumulation, driving to increased vascular permeability. Surprisingly, only 20% of patients with HAE-nC1-INH in Europe carry causal variants in the *F12* gene (17). Until 2018, only *SERPING1* and *F12* were known to cause HAE, which explains why HAE diagnosis has been mostly based on plasmatic determinations of complement proteins or the activity. The decreasing costs of Next Generation Sequencing (NGS) prompted the first whole-exome sequencing (WES) which allowed to detect variants affecting function in HAE-nC1-INH patients in angiopoietin (*ANGPT1*) and plasminogen (*PLG*) genes (18, 19). Using the same approach, Bork et al. also identified variants in the kininogen 1 (*KNG1*) gene as another cause of HAE (20). Most recently, two other causal genes were found by applying WES in HAE-nC1-INH families, encoding the heparan sulfate 3-O-sulfotransferase 6 (*HS3ST6*), and myoferlin (*MYOF*) (21, 22). The latter being causal strongly suggests a key role of VEGF-mediated signaling in HAE pathophysiology, although the mechanism triggering the symptoms remains unclear.

Because of the nonspecific signs, HAE remains a poorly recognized clinical entity, resulting in delayed diagnoses and deficient treatment conditions for long periods. In fact, a reported mean diagnosis delay of 7.9 years, ranging from months to 50 years, has been recently exposed in the HAE cases from the Canary Islands (23). Those patients lacking a diagnosis have an increased risk for morbidities and mortality compared to those that had been diagnosed, especially if attacks affect the airways (24). Advances in testing procedures and disease recognition have not improved HAE diagnosis, still representing a challenge for professionals (25). Currently, genetic testing is not part of the clinical diagnosis of HAE routine in many health care systems despite the updated HAE management guidelines recommend a genetic-based diagnosis in order to increase the diagnostic yield and reduce the diagnosis odyssey (11, 26, 27). Furthermore, the debilitating nature of HAE attacks makes early and precise diagnosis critical to rapidly establish the actions and treatments for short and long-term prophylaxis (28). Because of the importance of establishing an accurate diagnosis and to prescribe the optimal treatment, many countries have developed their own HAE patient registry. This has fostered the development of standards and specialized facilities to help managing and diagnosing patients while reducing the diagnosis delay. Here we present the first genetic characterization of HAE patients from the Canary Islands (Spain).

## Material and methods

### Patient population and setting

The study was approved by the Hospital Universitario Nuestra Señora de Candelaria (HUNSC; PI 57–17) Ethics Committee and written informed consent was obtained from all patients and relatives. The patient cohort is composed by affected individuals that had compatible clinical history of angioedema attacks without urticaria, and a differential diagnosis by blood molecular assays. We excluded patients using medication that were known to trigger angioedema attacks (i.e., angiotensin-converting enzyme inhibitors) and patients suffering other pathologies that can potentially cause angioedema attacks (i.e., hepatitis, HIV, hepatic/renal disorders, immunological deficiencies, and infections due to *Helicobacter pylori*).

HAE diagnosis was based on plasmatic determination of C1-INH and C4, and C1-INH activity, according to the international WAO/EAACI guidelines for the management of HAE (29).

### Whole-exome sequencing and variant calling

DNA was extracted from 4 mL of peripheral blood with Illustra™ blood genomicPrep kit (GE Healthcare; Chicago, IL). Alternatively, for pediatric patients and adults where blood sample extraction was not available, DNA from saliva samples were obtained with the OG-250 kit (DNA Genotek, Ontario, CA) following manufacturer’s instructions. DNA concentration was evaluated using the dsDNA BroadRange Assay Kit for the Qubit^®^ 3.0 Fluorometer (Termo Fisher Scientific, Waltham, MA).

**Figure 1:**
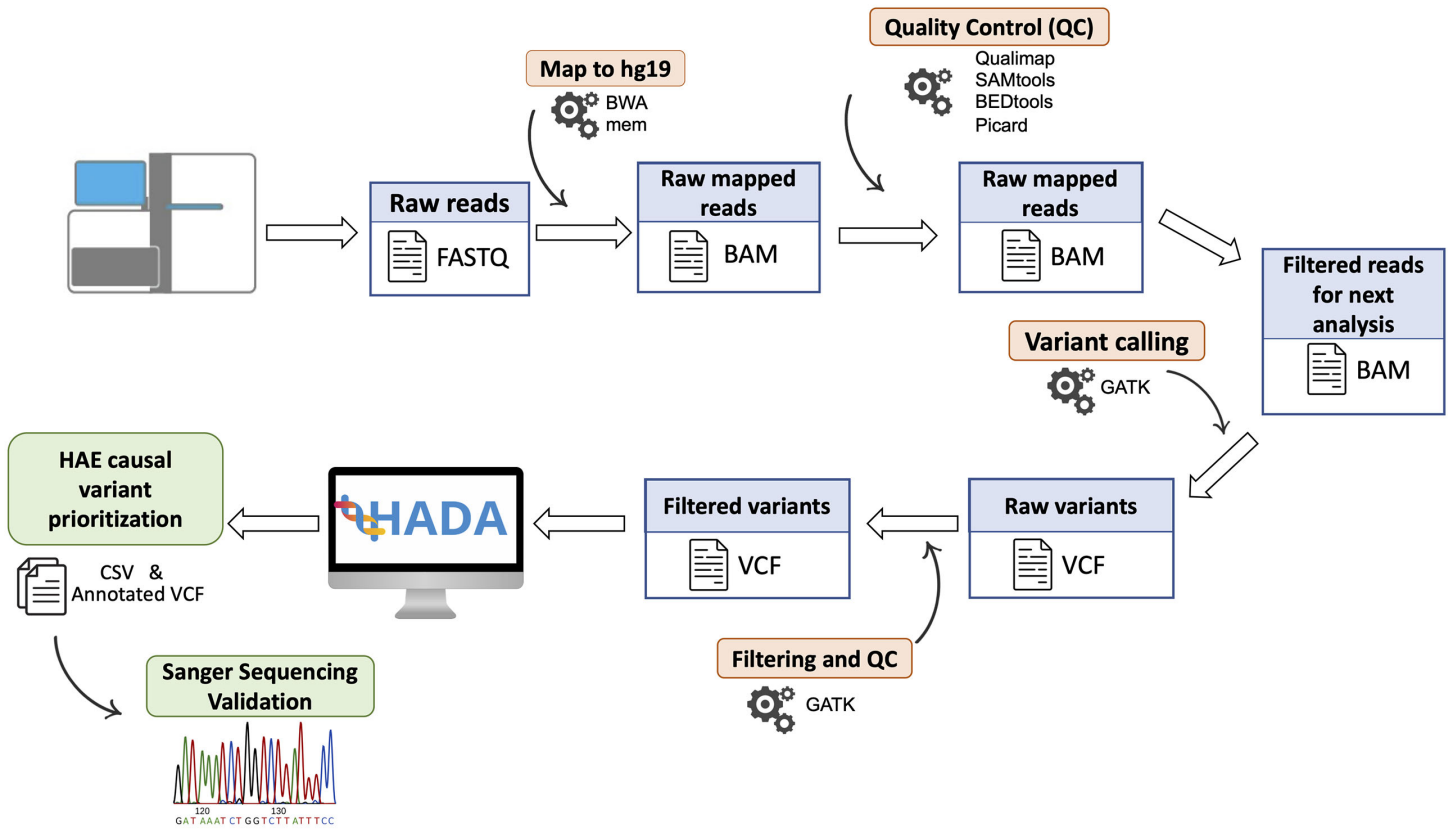
Schematic representation of the steps followed for patient DNA sequencing data. Algorithms are shown in orange and processed files in blue. The HAE causal variants prioritized by HADA and the Sanger validation step are shown in green.

Libraries were prepared using the TruSeq Rapid Exome Library Prep Kit (Illumina, San Francisco, CA). Library sizes and concentrations were assesed on a TapeStation 4200 (Agilent Technologies, Santa Clara, CA) and sequences were obtained with a HiSeq 4000 Sequencing System (Illumina, San Francisco, CA) with paired-end 75-base reads. PhiX was loaded and sequenced at 1% as an internal control of the experiments (**Figure 1**).

Sequencing reads were preprocessed with bcl2fastq v2.18 and mapped to hg19/GRCh37 with Burrows-Wheeler Aligner v0.7.15 (30), and BAM files were processed with Qualimap v2.2.1 (31), SAMtools v1.3 (32), BEDTools (33), and Picard v2.10.10 (http://broadinstitute.github.io/picard) for quality control steps. Variant calling of germline variants was performed using an in‐house bioinformatics pipeline based on the Genome Analysis Toolkit (GATK) v.3.8 (34). The pipeline has been designed for the detection of nucleotide substitutions (SNVs) and small indels (<50 bp) following the GATK best practices (35) and its description is publicly available (https://github.com/genomicsITER/benchmarking/tree/master/WES).

Subsequently, the identified variation was filtered by means of SAMtools and VCFtools (36) based on “PASS” filter, depth of coverage per position (≥ 20×), genotype quality (≥ 100), and mapping quality (≥ 50).

The analysis was carried out at the Teide-HPC Supercomputing facility (http://teidehpc.iter.es/en).

### Variant annotation and causal variant prioritization

The resulting variant calls were annotated with available information (**Figure 1**). For that, ANNOVAR (37) was used to include the allele frequency in reference populations, gene location, known functional consequences, links with disease based on ClinVar (38) and The Human Gene Mutation Database (39), and several pathogenicity scores including the Combined Annotation-Dependent Depletion (CADD, 40), among others. The classification of pathogenic potential of variants was obtained and annotated using InterVar software (41) following the American College of Medical Genetics and Genomics (ACMG) guidelines (42).

The annotated variant calls were individually processed for each patient by Hereditary Angioedema Database Annotation tool (HADA, http://hada.hpc.iter.es/), an in-house designed variant prioritization server of HAE genes to facilitate the identification of the variants affecting function as well as other accompanying information from the literature (13). In those patients where causal variants were not detected by HADA, Exomiser v12.1 was used to aim to identify the variants affecting function causing HAE (43). SQUIRLs v1.3.0 was used to specifically analyze candidate splicing variants and their predicted effect, prioritizing them by the internal pathogenicity score (44).

Finally, the variants that were classified as Variants of Uncertain Significance (VUS) that could be reclassified as Pathogenic/Likely Pathogenic variants based on complimentary information were validated by direct Sanger sequencing of PCR amplicons from the two strands. This assessment was performed by Macrogen Spain sequencing services with a customized design of primers (**Supplementary Table 1**).

### Assessment of potential structural variants in sequencing data

ExomeDepth v1.1.15 was used to identify putative structural variants (SVs) that could constitute possible variants affecting function causing HAE (45). Candidate variants obtained from BAM files were filtered to remove false positives calls by Bayes factor (BF) higher than 20.

To provide a clinical interpretation of the candidate SVs, filtered calls were processed with ClassifyCNV (46), based on the ACMG guidelines to determine pathogenicity potential. Complementarily, StrVCTURE (47) was used to distinguish pathogenic SVs from benign SVs that overlap exons based on CADD scores. The classification was carried out with the consensus of both algorithms.

### Screening for mobile elements in sequencing data

At least 17 *Alu* elements which are in *SERPING1* gene have been reported in the scientific literature. As such, pathogenic rearrangements associated with this repetitive element have been estimated to be responsible for approximately 15% of HAE cases (48). Because of that, the Soft Clipped Read Alignment Mapper v1.0.1 (SCRAMble) (49) was used to screen for mobile element insertions (MEIs) in the exome data, with a particular emphasis on *SERPING1*.

## Results

### Patient population and sequencing summary

Forty-one patients and nine healthy relatives from 29 unrelated families with HAE diagnoses residing in the Canary Islands were included in the study. Thirty-seven patients self-declared European ancestry, two declared ancestry from Colombia (family 3), and two from Israel (family 5). The study sample included eight males (19.5%) and 33 females (80.5%). The protective role of male hormones and the well-known influence of estrogens as one of the main triggers of HAE attacks could explain this sexual disbalance (50–52). The patients were aged between four and 72 years (mean: 36.8 ± 16.9 years) and a positive family history for HAE attacks was reported for 23 (56.1%) of them. Based on biochemical analysis, all HAE types are present in the study sample, where HAE-C1-INH was predominant, with 23 patients diagnosed for HAE type I (56.1%) and four patients for HAE type II (9.8%). The rest of patients (14, 34.1%) were diagnosed as HAE-nC1-INH, and most of them were females (92.8%).

WES of this case series yielded an average of 8.36 Gb per patient, with an average of 100% of on-target reads and a median depth of 57×, and a transition/transversion ratio in the range of 3.1 to 3.3.

### Causal variant identification and concordance between clinical and genetic assessments

**Figure 2:**
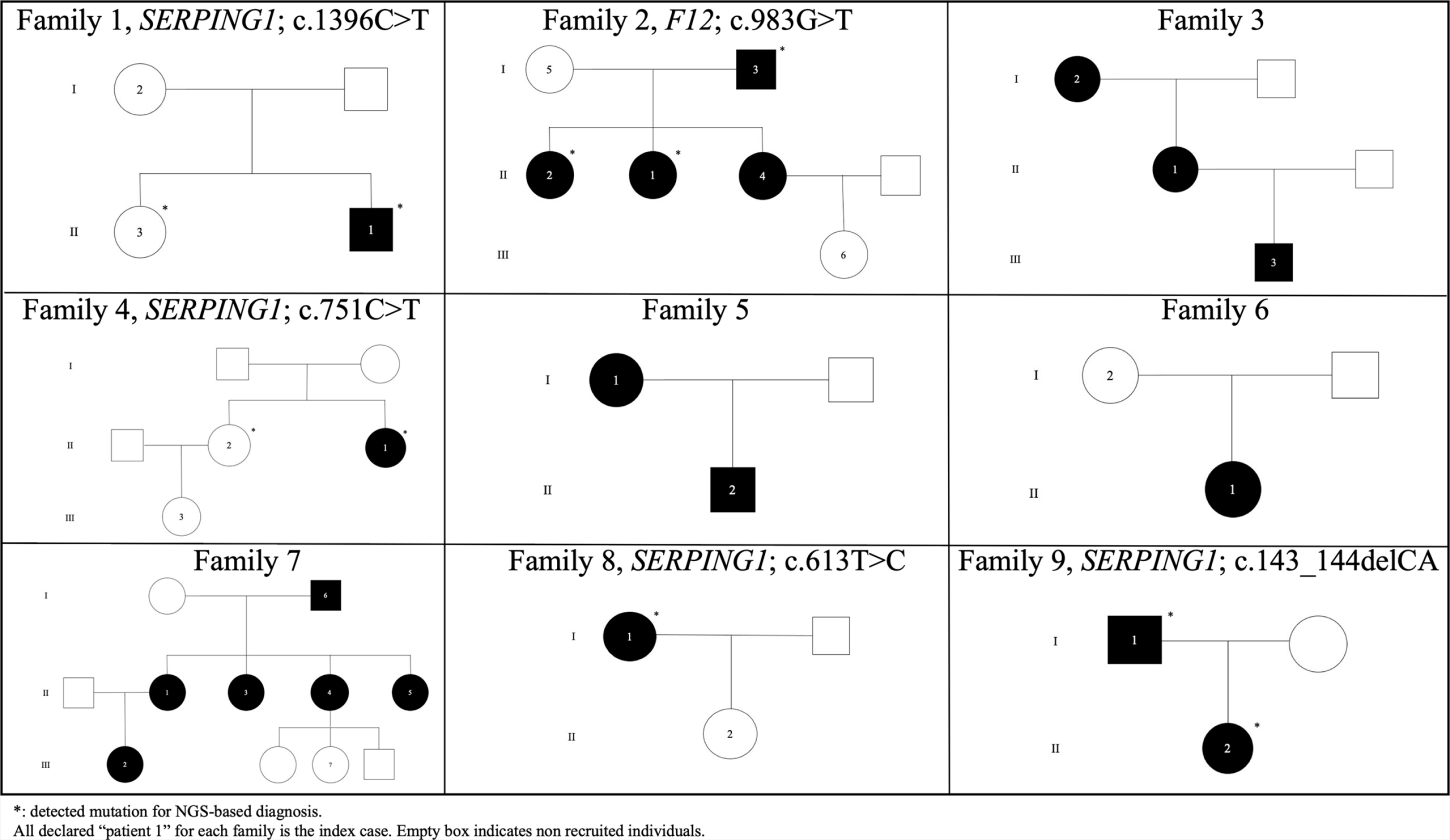
Pedigrees of unrelated HAE families in the Canary Islands.

Using HADA, we detected a total of six variants affecting function linked to different HAE types in the study (**Table 1** and **Figure 2**). *SERPING1* was the most commonly affected gene (n=5). Two unrelated families clinically classified as HAE type I were carriers of the synonymous variant affecting function c.751C>T; p.Leu251= (families 4 and 11), which was previously reported in Spain (53). The missense variant c.613T>C; p.Cys205Arg, previously described in Spanish cases, was also found in another family with HAE type I (family 8). We detected a likely pathogenic frameshift variant affecting function located in exon 3 of *SERPING1* gene, c.143_144delCA; p.Thr48SerfsTer9, which was associated with decreased C1-INH plasmatic levels and leading to HAE type I (family 9). We also detected the causative missense variant c.1396C>T; p.Arg466Ser in the index patient of family 1. In this family, the index patient [with normal levels of C1-INH (47 mg/dL), reduced C1-INH activity (10%) and low C4 levels (1.8 mg/dL) had similar biochemical findings as his asymptomatic sister (with normal) levels of C1-INH (65 mg/dl); reduced C1-INH activity (< 10%), and low C4 levels (7.6 mg/dl)]. Sequence analysis of this asymptomatic relative also releveled the missense variant c.1396C>T; p.Arg466Ser. This variant has been widely reported in Spanish patients in the literature, although linked to HAE type II instead (54).

**Table 1:** Detected causal variants affecting function of HAE in this study.

Family ID	Individual ID	Gene	Chr	Position start-end	HGVS	Aminoacid change	HAE type	ACMG class
**1**	1, 4	*SERPING1*	11	57381947- 57381947	c.1396C>T	p.Arg466Cys	II	Pathogenic
**2**	5, 6	*F12*	5	176831232- 176831232	c.983G>T	p.Thr328Lys	HAE-nC1-INH	Likely pathogenic
**3**	1, 2, 3	*SERPING1*	11	57373471- 57373471	c.686-12A>G	None	I	Likely pathogenic^†^
**4**	1, 2	*SERPING1*	11	57373548- 57373548	c.751C>T	p.Leu251=	I	Benign
**5**	1	*SERPING1*	11	57369570- 57369570	c.613T>C	p.Cys205Arg	I	Likely pathogenic
**9**	1, 2	*SERPING1*	11	57367442- 57367442	c.143_144delCA	p.Thr48SerfsTer9	I	Likely pathogenic
**10**	1	*SERPING1*	11	57378700-57378700	c.1100T>C	p.Leu367Pro	I	Likely pathogenic^†^
**11**	1	*SERPING1*	11	57373548- 57373548	c.751C>T	p.Leu251=	I	Benign

^**†**^Validated previously VUS reclassified according to publicly available genomic information.

Furthermore, a well-known causal variant of *F12* (c.983G>T; p.Thr328Lys) was detected in members of the family 2 with HAE-nC1-INH (**Figure 2**). This variant affecting function has not been previously reported in Spanish patients and is absent from ExAC (55) and gnomAD (56).

**Figure 3:**
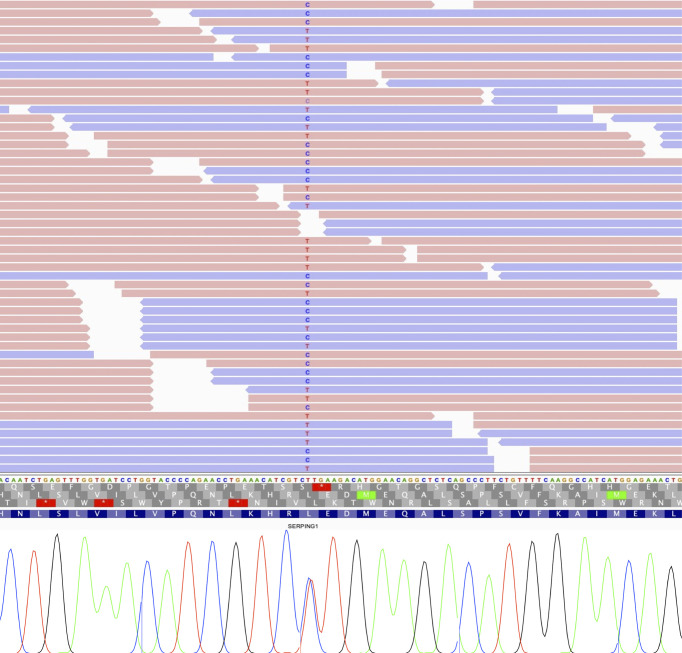
IGV view of sequencing reads supporting each allele of *SERPING1* variant (up), and Sanger sequencing validation showing both alleles (down).

We also identified a missense variant affecting function in *SERPING1* gene that was previously classified as a VUS. The variant c.1100T>C; p.Leu367Pro was detected in one patient for which information from relatives was lacking (**Figure 3**). Although this variant was described in previous genetic studies with HAE patients, it lacks population frequency data or prediction scores which could supporta a damaging effect, including individual predictions (SIFT, PolyPhen2, CADD, M-CAP, among others) and meta scores (MetaLR, MetaSVM, MetaRNN and REVEL). Despite that, we reclassified it as a likely pathogenic variant according to the ACMG guidelines using VarSome (57) based on the classification criteria PM1 (moderate), PM2 (moderate), PM5 (moderate), PP2 (supporting), and PP3 (supporting).

The manual inspection of sequencing data in the positions with candidate calls for SVs and MEIs revealed that all the prioritized findings were false positives due to low depth of coverage in the inspected regions, therefore, offering insufficient support to declare variants.

### Splicing defects detected

**Figure 4:**
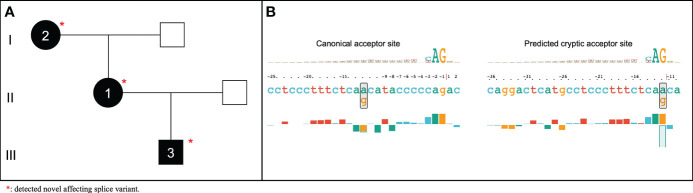
Pedigree of HAE affected family without previously reported causal variant **(A)** and representation generated by SQUIRLs of predicted acceptor site for the novel *SERPING1* c.686-12A>G affecting splice variant **(B)**.

The splicing variant defect c.686-12A>G in *SERPING1* was identified in all the affected patients of family 3 (**Figure 4**). This variant was previously reported in two unrelated Italian patients (58) and in one case of a Serbian HAE type I family (59). However, despite the authors did not assess the pathogenicity of the variant, its function was assessed using a minigene *in vitro* model (60). According to that study, the c.686-12A>G variant was found to provoke an aberrant splicing effect possibly triggering transcript degradation as deduced by the minimal amount of the transcript detected in the patient’s blood. Based on this extended information, we then scored this variant with the following ACMG criteria: BP4 (supporting), PS2 (strong), PM2 (moderate), PP1 (supporting), PP3 (supporting), and PP4 (supporting). As a result, VarSome reclassified the c.686-12A>G variant from VUS to Likely Pathogenic.

### Diagnostic yield

Aggregating all the described evidence for the variants affecting function detected across study patients from families with different affected members, we obtained a genetic diagnostic yield of 66.7% (95% Confidence Interval [CI]: 30.1-91.0%; 6/9 families) when considering only the previously known causal variants (**Figure 2**). However, the genetic diagnosis yield among proband-only cases decreased to 10.0% (95% CI: 1.8-33.1%; 2/20 cases). This finding supports the recommendation of the WAO/EAACI current guidelines for HAE management to recruit unaffected family members for causal variant screening.

## Discussion

In recent years, the widely use of NGS in the clinical field has significantly contributed to the achievement of early and accurate diagnosis. In this context, we aimed to characterize for the first time the underlying genetic causes of HAE in the Canary Islands, where clinical studies have estimated a prevalence of around 1.90:100,000 (23). This prevalence is higher than the that reported for overall Spain and is closer to that observed in other European studies, estimated in 2:100,000 (53). The isolation and the recent demographic history of the Canary Islands population could explain the HAE prevalence in the archipelago. Genetic studies have highlighted that the genes from the regulation of inflammatory response and the complement cascade are significantly enriched in the genomic regions that harbor distinctive genetic variation of this population (61). These findings, together with the estimated higher prevalence of HAE in the Canary Islands, may add to the underlying genetic factors involved in angioedema that have not been identified so far. In this context, NGS-based studies with HAE affected individuals and families are being constantly implemented in several countries with the aim of increasing the diagnostic yield, such as Denmark (62), Saudi Arabia (63), Turkey (64), Japan (65), Portugal (66), Greece (67), Norway (68), Switzerland (69), Croatia (70), China (71), Romania (72), Austria (73), and Puerto Rico (74), among others. However, NGS-based genetic studies have not been common in HAE studies until recently (17). Part of the explanation may reside in the fact that only two causal genes were identified until 2018 and that clinical diagnosis was essentially based on clinical symptoms and biochemical measurements of C4 and C1-INH levels/C1-INH activity in plasma (11). In certain cases, the clinicians could not refine the diagnosis in HAE borderline patients. In these cases, WES brings new and efficient opportunities to clearly identify the underlaying causes, especially in those without C1-INH deficiency (11). Besides, obtaining WES from all patients offers the possibility to unravel novel disease genes, as well as to identify the underlying causes in the known HAE genes in a single test. In this context, this study describes the first step in the genetic characterization of HAE patients from the Canary Islands. Subsequent studies based on phenotype-driven prioritizations of causal variants from the whole-exome and genome-wide studies will aim to identify novel genetic factors involved in HAE in this population.

Following the international WAO/EAACI guidelines for the management of HAE (26), we have obtained two remarkable results in one of the families. First, we amended the initial diagnosis based on the clinical and biochemical studies, which supported a HAE type I in family 1. However, the prioritization of genetic variants revealed a well-known variant of HAE type II (*SERPING1*; c.1396C>T). Secondly, this variant was also detected in one asymptomatic relative, allowing a diagnostic anticipation to the manifestation of symptoms in this family member. The development of bioinformatics tools focused on variant interpretation in the context of rare diseases accelerates diagnosis accuracy, which translates into a great benefit for patients and their families. However, we face a global problem with the high proportion of VUS detected in NGS-based studies, since it complicates an accurate clinical diagnosis identifying the genetic causes, as recommended by current international guidelines for HAE patient management. In addition, there is also a widespread conflict in the pathogenic classification rules for genetic variants. In family 4, we identified the synonymous variant *SERPING1*; c.751C>T, which has been classified as benign according to the ACMG guidelines. However, this variant has been declared as causal of the disease in Spanish patients with HAE (53). Synonymous variants can contribute to diseases, affecting gene function by splicing (75), binding of transcription factors (76), or mRNA stability (77), among others. They are common in the human genome, but their role in diseases have often been underestimated (78). It is well-known that variants affecting splicing are significant contributors to human diseases and are often missed in standard variant filtering approaches, which tend to focus on protein coding regions (79). In this study, we detected the *SERPING1* c.686-12A>G variant in all affected members of a recruited HAE type I family, previously reported as VUS and here reclassified as likely pathogenic. As indicated before, the increased number of sequencing studies in rare disorders have overcome the main limitations to the interpretation of the pathogenic potential of detected variants and their association with the disease (80, 81). In this context, the diagnostic yield of NGS-based solutions has improved to 35-60% nowadays (44). However, the genetic cause of most cases of this study is lacking (21/29 independent cases, 72.4%, 95% CI: 52.5-86.6). The diagnostic process of HAE is also a stressful period due to confusing signs with histaminergic angioedema attacks, which could be delayed for many years. To reduce the clinical odyssey, we previously designed HADA, a variant prioritization tool for DNA sequencing studies focused on HAE diagnosis (13). HADA retrieves previously reported causal variants in HAE patients in a few minutes, integrating extended genetic variant information from public updated databases used for pathogenic classification according to ACMG guidelines. Taken all prioritized variants by HADA in our study, we have estimated a NGS diagnostic yield of 66.7% (6/9 families). Unfortunately, the diagnosis yield decreases to 10.0% in proband-only analyses (2/20 cases). Remarkably, despite the capabilities of using a WES approach, we did not find variants affecting function in *PLG*, *ANGPT1*, *KNG1*, *MYOF*, and *HS3ST6* genes (involved in HAE-nC1-INH) in any of the studied families.

Our study has some strengths and limitations. Among limitations, we acknowledge that the number of patients where a causal variant was identified is limited, precluding for now a robust study of the relationships between phenotypes and genotypes, as well as the links with drug responses or the patient management. We also acknowledge a relatively low breath of coverage of some exons of the *F12* gene compared with that of *SERPING1* gene (66.49% of bases covered at least 10× in *F12* compared with 88.33% for *SERPING1*), which may hinder the identification of causal genetic variants in patients with clinical suspicion of HAE-nC1-INH. Another limitation was the low genetic diagnosis yield obtained among proband-only cases, which could be increased with the recruitment of family members for genetic causative variant identification and determining its origin (inherited vs *de novo*), as is recommended by WAO/EAACI guidelines. Although WES is not the optimal approach, we have aimed to identify and prioritize candidate SVs and MEIs. However, the results did not support the possibility that the cases could be caused by SVs in the known HAE genes. Even though HADA assist in the genetic testing of HAE patients, the tool was designed to prioritize genetic variants within the literature identified genes of HAE, thus lacking the function to identify novel HAE genes. To fill this gap, complementary studies relying on phenotype-driven approaches and genomic scans, among others, are needed to allow the identification of novel genetic factors of HAE, especially in those affected patients who remain without genetic diagnosis. Among the strengths of our study, we highlight the widespread recruitment of HAE patients and their relatives in the archipelago by the Allergy Services of the main hospitals from each island, allowing us to carry out an early detection of new cases of HAE and study the genetic causes through DNA sequencing. The detection of the *SERPING1* c.1396C>T; p.Arg466Ser variant led to a diagnosis anticipation in an asymptomatic relative considering both genetic results and the biochemical findings, allowing to achieve a precise diagnosis of HAE type II. Our study highlights the benefits of a first-tier genetic testing for HAE diagnosis and reinforces the need to clearly identify underlying genetic causes, as recommended by the international WAO/EAACI guidelines for the management of HAE and enhance the benefits of NGS as first-tier genetic testing tool for its diagnosis (26).

## Conclusions

To characterize the underlying genetic causes of HAE in the Canary Islands, we have performed the first genomic study of affected families from the region. A rapid detection of the causal variants in sequencing data from whole-exome was carried out based on HADA, providing in some families a refined diagnosis, and revealing the genetic affectation in asymptomatic members. Our results reinforce the necessity of NGS based studies on HAE patients to identify and characterize novel genetic factors involved in this disease. Complementary and more exhaustive analysis are required to identify the genetic cause in all the families.

## Data availability statement

The data presented in the study are deposited in the EGA repository (https://ega-archive.org/datasets/EGAD00001009318). Further inquiries can be directed to the corresponding author.

## Ethics statement

The studies involving human participants were reviewed and approved by Hospital Universitario Nuestra Señora de Candelaria Ethics Committee (HUNSC; PI 57–17). Written informed consent to participate in this study was provided by the participants’ legal guardian/next of kin.

## Author contributions

Conceptualization, CF and ACa; methodology, AM-A, ET-H and IM-R; patient and sample recruitment, LA-Q, EM-F, DM-B, EP-R, JG-R, and ACa; sample preparation and DNA sequencing, ACo, AI-C, and RG-M; formal analysis, AM-A, AM-B, LR-R, AA-G and JL-S; writing—original draft preparation, AM-A and CF; writing—review and editing, AM-A, IM-R, JL-S, ACa and CF; supervision, CF; funding acquisition, ACa, IM-R and CF. All authors have read and agreed to the published version of the manuscript. All authors contributed to the article and approved the submitted version.

## Funding

This work was supported by the Ministerio de Ciencia e Innovación (RTC-2017-6471-1; AEI/FEDER, UE) and the Instituto de Salud Carlos III (CD19/00231), which were co-financed by the European Regional Development Funds ‘A way of making Europe’ from the European Union; Cabildo Insular de Tenerife (CGIEU0000219140); Agreement OA17/008 with the Instituto Tecnológico y de Energías Renovables (ITER) to strengthen scientific and technological education, training, research, development, and innovation in genomics, personalized medicine, and biotechnology; Fundación Canaria Instituto de Investigación Sanitaria de Canarias (PIFIISC19/48); and SEAIC Foundation (18_A01). A.M.-A. and E.T.-H. were supported by a fellowship from Agencia Canaria De Investigación Innovación Y Sociedad De La Información Gobierno De Canarias (TESIS2020010002 and TESIS2021010046, respectively) co-funded by European Social Fund. AAG was supported by Ministerio de Universidades (modality Margarita Salas). The content of this publication is solely the responsibility of the authors and does not necessarily reflect the views or policies of the funding agencies.

## Acknowledgments

We would like to thank the patients and nurses involved in the design of this study. AM-A, ET-H, AM-B, and LR-R. acknowledge the University of La Laguna for the training support during their PhD studies.

## Conflict of interest

The authors declare that the research was conducted in the absence of any commercial or financial relationships that could be construed as a potential conflict of interest.

## Publisher’s note

All claims expressed in this article are solely those of the authors and do not necessarily represent those of their affiliated organizations, or those of the publisher, the editors and the reviewers. Any product that may be evaluated in this article, or claim that may be made by its manufacturer, is not guaranteed or endorsed by the publisher.
