# Supplementary material for: A catalog of the genetic causes of hereditary angioedema in the Canary Islands (Spain)
Source: Front Immunol. 2022 Sep 20;13:997148. doi: 10.3389/fimmu.2022.997148 (PMC9531158; doi:10.3389/fimmu.2022.997148)
Supplement: Supplementary file 1 [file Table_1.docx]

Supplementary Material

# Supplementary Figures and Tables

Supplementary table 1. Setting conditions and primers design for Sanger sequencing and validation of novel candidate causal variants.

| Target variant | Primer sequences | Amplicon length (bp) | Annealing temperature (ºC) | ACMG reclassification |
| --- | --- | --- | --- | --- |
| *SERPING1*;c.1100T>C | CAGGAGAGAGATGCGGTAGG | 404 | 60 | Likely pathogenic^†^ |
|  | TCCTGCAACCCTGCAAGTTA |  |  |  |
